# Perspective: Assessing the Flexible Acquisition, Integration, and Deployment of Human Spatial Representations and Information

**DOI:** 10.3389/fnhum.2018.00281

**Published:** 2018-07-11

**Authors:** Michael J. Starrett, Arne D. Ekstrom

**Affiliations:** ^1^Department of Psychology, University of Arizona, Tucson, AZ, United States; ^2^Department of Psychology, University of California, Davis, Davis, CA, United States; ^3^Center for Neuroscience, University of California, Davis, Davis, CA, United States; ^4^Neuroscience Graduate Group, University of California, Davis, Davis, CA, United States

**Keywords:** spatial representations, spatial information, navigation, egocentric, allocentric, virtual reality, relative vector discrimination (RVD) task

## Abstract

Studying human spatial navigation in the lab can be challenging, particularly when including non-invasive neural measures like functional magnetic resonance imaging (fMRI) and scalp encephalography (EEG). While there is broad consensus that human spatial navigation involves both egocentric (self-referenced) and allocentric (world-referenced) coding schemes, exactly how these can be measured in ecologically meaningful situations remains controversial. Here, we explore these two forms of representation and how we might better measure them by reviewing commonly used spatial memory tasks and proposing a new task: the relative vector discrimination (RVD) task. Additionally, we explore how different encoding modalities (desktop virtual reality, immersive virtual reality, maps, and real-world navigation) might alter how egocentric and allocentric representations manifest. Specifically, we discuss desktop virtual reality vs. more immersive forms of navigation that better approximate real-world situations, and the extent to which less immersive encoding modalities alter neural and cognitive codes engaged during navigation more generally. We conclude that while encoding modality likely alters navigation-related codes to some degree, including egocentric and allocentric representations, it does not fundamentally change the underlying representations. Considering these arguments together, we suggest that tools to study human navigation in the lab, such as desktop virtual reality, provide overall a reasonable approximation of *in vivo* navigation, with some caveats.

## Introduction

**FIGURE 1:**
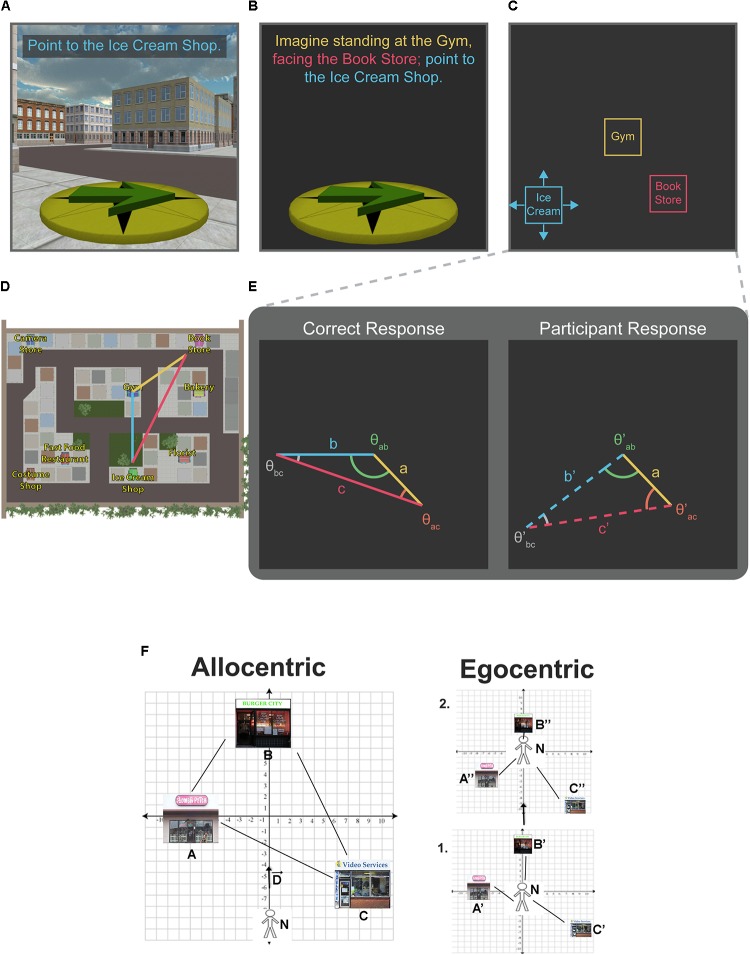
Pointing tasks. Single-trial examples for computer versions of the scene and orientation-dependent (SOP) pointing task **(A)**, the judgments of relative direction (JRD) pointing task **(B)**, and the proposed relative vector discrimination (RVD) task **(C)**. In each example, participants’ memory for the location of the Ice Cream Shop (blue text) is being tested. Example trials are based on the virtual environment used by Starrett et al. (in press) **(D)**. Unlike the JRD and SOP tasks, which only yield angular precision estimates, the RVD task yields both angular and distance information [note that the anchor vector (yellow line) is common across the correct and participant response, and the placement of the target store establishes the remaining two legs of a triangle (red and blue lines)] **(E)**. A reproduction of Figure 1 from Ekstrom et al. (2017) shows the cartesian relationship between allocentric (left panel) and egocentric (right panel) as a displacement vector from the navigator **(F)**.

Cognitive neuroscience provides a wide variety of behavioral and neural tools to assay cognitive processes and neural systems that underlie human spatial navigation. However, like any measurement tool in science, there are limitations to how they can be applied and exactly what information they provide. In terms of behavioral measures for spatial knowledge, two pointing tasks have been widely used: the scene and orientation-dependent pointing (SOP) task and the judgments of relative direction (JRD) pointing task. In the SOP task, shown in **Figure 1A**, participants are oriented within the environment and then asked to point to target objects. In the VR version, all targets are removed, and background information provides visual orienting information (e.g., Zhang et al., 2014). In the real-world version, participants are blindfolded but oriented within the environment via body-based input (e.g., Wang and Spelke, 2000). Conversely, the JRD task is conducted with participants either disoriented within the environment, or moved to a different environment, where they are provided a triad of targets (delivered via text or by the experimenter, verbally; “Imagine standing at A, facing B; point to C."). The first two targets serve to established an imagined heading to orient to while participants point to the third target in the triad (Shelton and McNamara, 2001; Mou et al., 2004; Waller and Hodgson, 2006; Starrett et al., in press). An example trial is shown in **Figure 1B**. There are systematic differences in what is measured by each task, and both tasks have limitations related to exactly what they measure in terms of underlying spatial representations (Mou et al., 2004; Kelly et al., 2007; Ekstrom et al., 2014; Zhang et al., 2014).

In terms of neural assays, functional magnetic resonance imaging (fMRI) requires participants to lay supine while navigating in virtual reality (VR), and challenges remain for conducting scalp EEG during real-world exploration (but see Gramann et al., 2010). Our main focus in this perspective is therefore to consider the limitations imposed by the SOP and JRD tasks and studying navigation in VR more generally. We also consider the valuable information we can nonetheless glean from them in terms of how we navigate and suggest a new relative vector discrimination (RVD) task (see **Figure 1**) aimed to better describe spatial memory for allocentric reference frames and the flexibility of representations across various spatial task demands.

**FIGURE 2:**
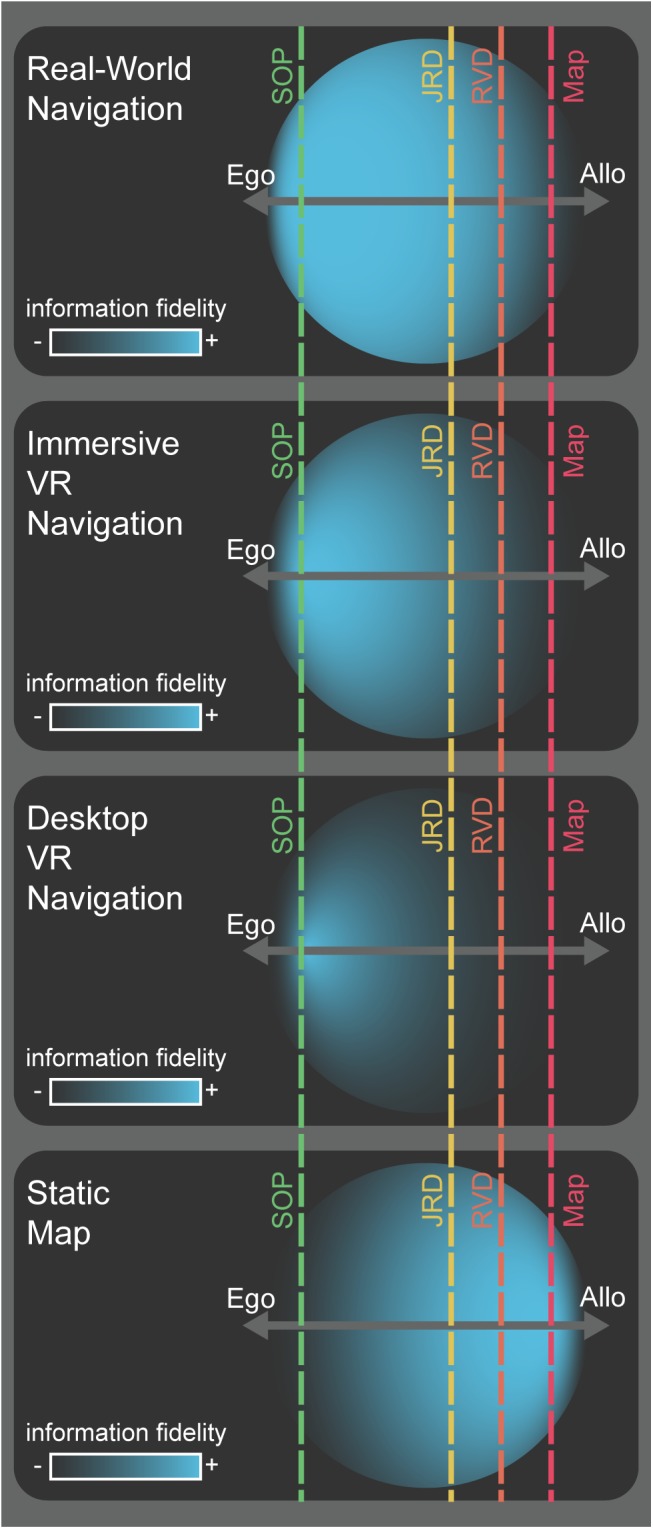
Conceptual model for the encoding and deployment of spatial information. Hypothetical spatial information (blue circles) acquired during early encoding of a novel environment via various encoding modalities (labels, left). Each example shows the fidelity of spatial information acquired (density and center of mass for blue circles) along an egocentric-to-allocentric continuum (dark gray, horizontal axes). The extent to which the circles project outward from the egocentric-allocentric axis illustrates information that, while not strictly spatial in nature (e.g., semantic or episodic memory), can be used to derive spatial information or drive inferences about the environment. The offset of the vertical, dashed, lines from the center of the egocentric-allocentric axis represents the hypothetical proportion to which that task depends more on egocentric or allocentric reference frames for its optimal solution (SOP: green, JRD: yellow, RVD: orange, map drawing: red).

One way to consider the relative demands of the SOP and JRD tasks is along an egocentric to allocentric continuum (see **Figure 2**), which also allows us to consider how different levels of immersion in VR might affect where they fall on this spectrum. We suggest that future experiments should focus on how spatial information manifests and is accumulated during various encoding modalities (e.g., route versus map learning). Separately, we consider how this information might be strategically deployed depending on flexible task demands during retrieval (e.g., SOP, JRD, map drawing, etc.). We can potentially better model and understand the nature of representations underlying human spatial navigation by considering how spatial information is first encoded along the egocentric to allocentric continuum, and then subsequently retrieved depending on the task demands.

## The Egocentric to Allocentric Spectrum: Where Do the Different Pointing Tasks Sit?

One of the most widely used distinctions in spatial navigation involves the idea of two fundamentally different forms of representations, egocentric vs. allocentric. Briefly, navigation involving an egocentric representation employs a coordinate system referenced to one’s current position and facing direction and is most familiar in navigating our immediate, peripersonal space. Examples include knowing where a chair is in front of us so that we can avoid colliding with it when we get up or reaching for a mug next to us. One disadvantage of egocentric coordinates, however, is they change constantly as the navigator moves, requiring continuous updating of one’s position. In contrast, an allocentric representation involves reference to objects that remain constant in the external world. For example, using other landmarks to remember how to get to a goal. A disadvantage of an allocentric representation is that it requires reference, and memory for, multiple landmarks.

There is at least some evidence that the SOP task assays primarily egocentric forms of spatial representation while the JRD task assays primarily allocentric forms of representation (**Figure 2**). In particular, there is general agreement that the SOP task is primarily egocentric, provided that participants are oriented when pointing and that the dependent measure is absolute pointing error (Wang and Spelke, 2000; Holmes and Sholl, 2005; Waller and Hodgson, 2006; Zhang et al., 2014). In contrast, there is significantly less agreement regarding the JRD task and the extent to which it provides primarily allocentric, or some complex combination of egocentric and allocentric, information (Ekstrom et al., 2017). Specifically, given that imagined, first-person headings are a fundamental part of the task (“imagine you are facing X”), it seems difficult to fully discount the contributions of egocentric viewpoint information from JRDs (Ekstrom et al., 2014). Indeed, previous work has demonstrated a bias to perform better when pointing at targets in the forward hemifield of the imagined heading and that this bias is weaker or absent when information is learned from a map (Sholl, 1999; Kelly and McNamara, 2009), suggesting that spatial information is either acquired or deployed differently across learning modality even for the same retrieval task. In addition, a recent article by Wang (2017) demonstrated that tasks like the Morris Water Maze, often argued to rely on allocentric representations (Morris et al., 1986), can also be solved using egocentric coordinates (Wang, 2017). Finally, the JRD task does not involve an estimate of distance, an important component of allocentric representation more generally (because egocentric representations are more likely to involve viewpoint and bearing-dependent “snapshots,” distance is likely less relevant).

## The Relative Vector Discrimination (RVD) Task: A More Allocentric “Allocentric” Task

We propose a new RVD spatial memory task to provide a fundamentally more (although still not purely) allocentric task than the JRD task and thus a better foil for the SOP task (see **Figure 1**). Inspired by the task used by Han and Becker (2014), the RVD task is framed in the third-person, with participants receiving a top–down view of the locations of two target stores and placing a third freely (**Figure 1C**). In the RVD task, participants are presented with a blank screen showing two “anchor” landmarks positioned relative to one another on the screen. The position of these landmarks on the screen is fixed. Depending on the vector defined by the positions of the anchor landmarks, participants will be required to place a third target landmark on the screen relative to the anchor landmarks (**Figure 1C**). One benefit of the RVD task is that it provides a measure of both angle and distance, as well as latency, within the same task. Error can be quantified by comparing the geometry of the triangle created by the anchor vector and the correct placement vectors with the anchor vector and the participant’s placement vectors (**Figure 1E**).

While the RVD task differs in several important ways from the SOP and JRD, performance on all three tasks is dependent on similar spatial memory principles such as environmental geometry, salient landmarks, and learned viewpoints or routes (depending on learning modality). For example, the anchor vector in the RVD task can be thought of as an analog to the imagined heading in JRD task or the oriented perspective in the SOP task. Therefore, the same types of independent manipulations can be applied to the RVD task (e.g., alignment with learned perspectives or environmental geometry). In fact, as suggested by the title of this section, the primary objective in creating this task is to yield a dependent variable comparable to that of the JRD, but with intrinsically more allocentric demands based on how the task is framed. This is illustrated in **Figures 1A–E**, where the orientation, imagined heading, and anchor vector in the SOP, JRD, and RVD, respectively are identical. The linearly transformable relationship between egocentric and allocentric cartesian coordinates is also illustrated in Figure 1 of Ekstrom et al. (2017), shown in **Figure 1F**. Therefore, if these tasks were identical, the triangles derived from responses on any of them should theoretically be geometrically similar (i.e., the corresponding angles should be equal), if not identical. Any deformation would be indicative of differences in task demands. Notably, with the inclusion of distance estimates in the RVD, other metrics comparing the deformation of entire shapes may be used to compare triangles derived from participants’ responses against those derived from the correct response, such as those put forward by Basri et al. (1998).

When implementing the RVD task, there are several important parameters and aspects of the task to consider. (1) The degree of potential egocentricity can be manipulated even within the parameters of the RVD task (further supporting the idea of flexible spatial demands for retrieval tasks). For example, the anchor vector could be constrained to always originate from the center of the screen (as in the example trial shown in **Figure 1C**). While this may prove useful for experimental designs that require fixation, such as fMRI or eye tracking paradigms, doing so also limits the extent to which experimenters can rule out participants’ reliance on a central “self-” centered anchor within their visual field. Other parameters that may affect the egocentric versus allocentric demands of the RVD task include length of the anchor vector relative to a learned viewpoint (particularly for map learning), the orientation of the anchor vector relative to learned viewpoint (global map orientation for map learning or possibly initial or final viewpoints during navigation), and potentially others. (2) Whether RVD performance is being compared with performance on other spatial memory tasks such as the SOP, JRD, or map drawing. In the case of the SOP and JRD tasks, which traditionally do not include distance estimates, it may prove beneficial to constrain the anchor vector to originate from the origin (as in **Figure 1C**) to provide a clear comparison angle and analog for imagined heading used in the SOP and JRD. Additionally, the length of the anchor vector could be constrained either to be identical on every trial or scaled to corresponding lengths relative to the geometry of the environment if the experimenter wishes to attempt to control for angular biases related to distance. Ultimately, the consideration of these parameters will depend on the design of the experiment and the questions being addressed (Waller and Hodgson, 2013).

The RVD task thus provides an additional position along the egocentric to allocentric continuum of spatial information (see **Figure 2**) that can be probed during recall. This will allow future experiments to expand on previous findings comparing the JRD and SOP tasks (Waller and Hodgson, 2006; Zhang et al., 2014) by observing and contrasting performance on the RVD task across encoding modalities (routes/maps) relative to the JRD and SOP tasks. We hypothesize that the RVD task can be used to coerce the deployment of more allocentric spatial information in well-learned environments or the conversion of egocentric information to make inferences from an imagined or low-fidelity allocentric reference frame (**Figure 2**), consistent with previous suggestions regarding how participants often utilize allocentric representations (Mou et al., 2006; Newman et al., 2007).

## The Space Between Reference Frames: Encoding Modalities, Retrieval Demands, and How They Interact with Spatial Knowledge

As suggested by the BBB model (Byrne et al., 2007) but worked out in detail in a recent computational paper (Wang, 2017), the primary difference between egocentric and allocentric representations involves keeping track of one’s displacement (**Figure 1F**; reproduction of Figure 1 from Ekstrom et al., 2017) (see also Ekstrom and Isham, 2017, **Figure 1**; Wang, 2017, **Figure 2**). As described earlier, in an egocentric reference frame, the coordinates for one’s position stay constant while those for landmarks continuously change. In contrast, in an allocentric reference frame, the positions of landmarks stay constant while those of the self continuously change. Thus, the allocentric reference seems computationally more efficient because only the movement of the navigator needs to be maintained, and thus eventual conversion of egocentric to primarily allocentric coordinates would appear advantageous. Consistent with this idea, during navigation of well-known environments, participants appear to prefer allocentric reference frames, but when allocentric information is not reliable or is of low fidelity, egocentric reference frames dominate (Mou et al., 2006; Newman et al., 2007).

While it is possible to define and distinguish egocentric and allocentric reference frames mathematically and anecdotally, and why an allocentric reference in particular might be most advantageous for navigating, in practice, the interaction and dynamic use of either or both can be difficult to parse, particularly given that the main difference involves a simple linear transformation (adding/subtracting one’s displacement). Consider the example of driving with a global positioning system (GPS). In this scenario, it is unlikely that the GPS or the driver’s view of the road will be used in isolation. Most likely, attention will constantly shift from the road to the GPS and back, all the while updating and integrating information from each source. This example illustrates how navigation in the modern world rarely involves a static egocentric or allocentric reference frame.

Moreover, the specific reference frames used may not be purely egocentric or allocentric. While GPS devices do show a map view of the environment, this map is often updated such that an icon indicating the user’s current position is constantly centered and sometimes even facing the current direction of travel, introducing an egocentric element. The GPS represents an example of a hybrid reference frame that may be integrated with, translated to, or even represented independently from more egocentric and allocentric reference frames as one navigates (Trullier et al., 1997; Eichenbaum, 2017). Such hybrid information could facilitate more rapid integration with real-world egocentric information in lieu of actual topological or survey knowledge by placing the onus of any computational conversions or representations on the GPS rather than areas proposed to be important for egocentric to allocentric conversion like retrosplenial cortex (Burgess, 2006; Byrne et al., 2007; Epstein, 2008; Ekstrom et al., 2017).

In **Figure 2**, we present a conceptual model for describing spatial information along the egocentric-to-allocentric continuum. Spatial information is indicated by the blue area of the circle for which density denotes more high-fidelity information and the center of mass shows the utility of that information for task demands ranging from egocentric to allocentric. The position, density, and dispersion of the spatial information circles are largely influenced by learning modality (illustrated by contrasts between panels in **Figure 2**), but for both egocentric and allocentric information, participants acquire varying amounts and fidelity in each learning condition. Various retrieval task demands are depicted by vertical, dashed lines, with the offset from the center of the continuum illustrating the relative egocentric-allocentric dependence of the task. As emphasized in the figure, not only are the learning modality and retrieval task demands critical individually, but so is the interaction between two. Here, spatial information is represented by a 2-dimensional circle along a 1-dimensional egocentric-allocentric axis. The extent to which the circle projects outward, orthogonally, from the egocentric-allocentric axis is intended to represent memory or knowledge that may not be specific to the environment being learned, like semantic (many cities are arranged in blocks or grids) or episodic memory (a car almost hit me when I crossed that street once). Thus, this conceptual model attempts to account for not only spatial information categorized from the “primary” reference frame, but also from the non-dominant reference frame and more abstract information like Bayesian priors or heuristics.

The model illustrates several important properties of how we encode and deploy spatial information, which can be impacted by (1) the encoding modality (e.g., routes or maps), (2) the strength or fidelity of the spatial information encoded (also partially dependent on the encoding modality), (3) the optimal reference frame used for solving a specific spatial memory task (note that none of these tasks can guarantee how participants will solve a retrieval task, rather only encourage selection of a desired, optimal solution), and (4) the ability to deploy prior knowledge and heuristics to make inferences using one reference frame when spatial information is encoded primarily from the other reference frame.

Here, we define heuristics as “up is north” (Brunye et al., 2012) or the well-described advantage that comes with remembering facing locations aligned with the axes of a rectangle compared to misaligned (e.g., Mou and McNamara, 2002; Starrett et al., in press). In the case of using rectangles as a heuristic, memory for the location of a target can be bound to the geometry of the rectangle, in that any points defined by its orthogonal distance to each side of the rectangle share the same principle axes relative to the environment. The selection of a singular, bipolar axis or two primary axes, akin to cardinal directions on a map, is consistent with how we often learn from and interpret maps and is thus a familiar and efficient way to remember any space with rectangular properties. In terms of our model, this would involve using information in denser areas or from other spatial representations to fill in less dense areas (**Figure 2**), perhaps temporarily while more high-fidelity information is acquired to establish that reference frame. In this way, our model helps explain several previously described phenomena in the human spatial navigation literature that lack a clear theoretical connection with egocentric vs. allocentric representation.

## How Egocentric and Allocentric Representations Interact with Encoding Modality

Understanding the neural basis of egocentric and allocentric representations is an important research endeavor (for recent review, see Ekstrom et al., 2017). One important limitation inherent in most neural recordings from humans is that they have traditionally been limited to desktop interfaces, which lack many of the characteristics of real-world navigation. Namely, desktop VR does not provide idiothetic, self-motion cues because participants sit stationary, and desktop VR may introduce conflict between real-world and virtual allothetic cues. Even the rendering of optic flow, while still 3-dimensional, lacks the exact stereoscopic immersion of real-world experiences (see Snow et al., 2014). Specifically, in the context of navigation as a means for encoding spatial representations, the removal of such self-based information could fundamentally alter the neural processes and mechanisms being studied under such conditions relative to real-world (for review, see Taube et al., 2013). For example, desktop VR lacks true head-turns, resulting in little or no vestibular information during such navigation tasks. Vestibular lesions have been shown to significantly alter hippocampal theta oscillations in rats (Russell et al., 2006), an important neural signal related to spatial navigation, raising the possibility that the lack of head-direction input could fundamentally alter these codes. In humans, diminished vestibular information relative to real-world navigation may have downstream effects on other types of spatial processing neurons such as path cells, boundary-vector cells, or head-direction cells (Ekstrom, 2010; Jacobs et al., 2010; Taube et al., 2013).

To what extent does the lack of head-direction input limit the nature of spatial representations that can be assayed with desktop VR in humans? Invasive recordings of the hippocampus in humans, monkeys, and rats have all identified place cells, with Miller et al. (2013) showing that during later free recall of items associated with locations in a virtual environment, the same or nearby hippocampal place cells fired. These findings suggest that desktop VR, and even desktop presented stimuli in the absence of immersive scene information, do capture sufficient information to recapitulate neural codes from the real world. Notably, view-coding cells are also present in both monkeys and humans, suggesting a specific mechanism that could favor view-dependent, VR-based navigation (Ekstrom, 2015). Thus, the presence of place cells across modalities and species as well as view-coding mechanisms in primates, argue against the idea that the lack of explicit head-direction input during desktop VR somehow fundamentally changes how we code space during navigation.

Similarly, low-frequency, movement-related theta oscillations in the hippocampus, semi-periodic fluctuations in the local field potential that manifest during navigation, are present during desktop VR (Watrous et al., 2011, 2013; Bohbot et al., 2017), retrieval of spatial information (Ekstrom et al., 2007), encoding and retrieval of verbal associations (Sederberg et al., 2003; Yaffe et al., 2014), and during real-world navigation (Aghajan et al., 2017; Bohbot et al., 2017). One possibility is that the frequency of theta oscillations during real-world navigation in humans might be higher than VR, similar to higher frequency theta oscillations in rodents (Yassa, 2018). This in turn might seem to bolster the argument that VR and real-world navigation alter underlying neural representations (Aghajan et al., 2017). It is important to note, however, that the wireless hippocampal recordings used by Aghajan et al. (2017) could not detect oscillations below 4 Hz due to hardware-enforced, bandpass filtering. Indeed, Bohbot et al. (2017) used wired recordings during free ambulation and analyzed frequencies as low as 1 Hz, finding that low frequency oscillations were present across the range of 1–12 Hz, with only a slight difference in frequency across all electrode recordings for VR vs. real-world movements (see also Aghajan et al., 2017, Supplementary Figure 4 for examples from their wired recordings). Thus, low-frequency hippocampal theta oscillations, an important navigation-related neural signal, are present during a variety of immersed and non-immersed memory and navigation tasks to comparable extents, all of which appear sufficient to induce its presence.

If the lack of vestibular and other whole-body input did dramatically affect our underlying spatial codes, we might expect significant changes in how we learn environments with a full range of body-based cues compared to an impoverished set, such as navigating in desktop VR. Past behavioral studies have investigated these issues, with one early study suggesting that VR learning transfers only minimally to real-world environments (Kozak et al., 1993). A later study, however, by Richardson et al. (1999) only observed diminished performance when pointing tasks required participants to remember spatial relationships from different floors of a virtual building. One major issue with these early studies, however, is that VR technology was in its relative infancy and the complexity of visual displays and environmental geometry were relatively limited. With VR capturing real-world environments to only a limited extent, it is not surprising that transfer was minimal.

Recent experiments have begun to test learning in VR under a richer set of conditions than simply comparing desktop VR to real-world navigation, and in particular, the advent of the head-mounted display has allowed researchers to render complex visual environments as a participant freely ambulates within it. In one set of experiments participants had to search virtual birdhouses for a target. Importantly, they tested participants either while standing still and using a controller for rotations and translation, standing still but physically rotating while using a controller for only translations, or physically walking and turning. Results showed that physically walking and turning with the head-mounted display, i.e., the combination of vestibular, proprioceptive, and somatosensory information, significantly improved performance (Ruddle and Lessels, 2006, 2009) compared to the other conditions; thus, vestibular input, on its own, did not seem to be as important as the combination of multimodal cues.

More recently, Chrastil and Warren (2013) used a hedge-maze navigation task and assessed performance based on the later use of shortcuts to target locations. Participants learned the maze either by watching a video, being moved through the environment in a wheelchair, or by walking. Performance in the wheelchair condition, which provided rich vestibular information, was statistically equivalent to performance in the video condition, which was slightly above chance. In contrast, performance was better in the walking condition, which provided vestibular and proprioceptive input, than video condition. Other studies aimed at dissociating the contributions of proprioception (walking) and vestibular (turning) information have found that rotational information alone contributes minimally to performance on spatial estimates (Ruddle et al., 2011). While other studies have yielded conflicting results with regard to the importance of translational versus rotational body-based cues (see Chance et al., 1998), one common finding across these studies is that the full-range of multimodal body-based input seems to boost performance during navigation with the majority of findings suggesting that vestibular input alone is not necessary for the normal expression of spatial representations in humans. The question becomes even more complex when accounting for the plethora of ever-evolving interfaces used for immersive interaction with large-scale virtual environments, (head-mounted displays, CAVEs, omnidirectional treadmills), which introduce further nuances to the information available for encoding during spatial learning and should be considered when developing navigation and spatial learning paradigms (for a somewhat recent review, see Waller and Hodgson, 2013; see also: He et al., 2017; Paris et al., 2017; Starrett et al., in press).

While it is clear that there are some behavioral differences between learning with the full-range of body-based input vs. an impoverished set of cues, importantly, at least some of this may be attributable to memory-related effects (Tulving and Thomson, 1973). In other words, when we have a richer set of cues to encode information in the first place, we will benefit from these multiple cues during retrieval to a greater extent than when encoding and retrieval occur with a smaller number or different set of cues. A greater number of modalities also mean that different learning systems can work in parallel, providing the potential for faster learning. Thus, we believe that the evidence argues strongly against the idea that vestibular input is necessary for “normal” spatial navigation, by which we intend to say that navigation derived from visual input alone is largely sufficient for the types of modality independent spatial representations we form during navigation (see Waller and Hodgson, 2013). Nonetheless, vestibular (and other body based) cues clearly serve to enhance the fidelity of these representations (Klatzky et al., 1998). In this way, modality independent navigational representations can operate largely in the absence of vestibular cues, although such representations are more flexible and enriched in the presence of modality-dependent forms of representations involving vestibular and other body-based cues (Wolbers et al., 2011).

Another issue to consider is passive vs. active engagement with the environment. Past behavioral studies have provided mixed results regarding whether active navigation results in better performance on spatial tasks like map-drawing and shortcut-taking (Chrastil and Warren, 2012). Neurally, active engagement with stimuli alters neural codes in brain areas important to navigation and memory like the hippocampus (Voss et al., 2011). Even here, though, hippocampal mechanisms like pattern completion/separation (Stokes et al., 2015) still operate under conditions of passive navigation. It remains to be determined, then, the extent to which active vs. passive navigation significantly alters navigation-related neural coding in humans. Furthermore, while we have described how encoding modality might affect spatial representations more generally, exactly how it might differentially affect egocentric vs. allocentric representations both neurally and cognitively remains largely untested. We hope, however, that our discussions above, and **Figures 1**, **2**, will provide some possible theoretical predictions for testing these issues in the future.

## Conclusion

To simplify the complexity of our considerations in cognitive neuroscience, it is often helpful to reduce the cognitive processes under consideration to more “elemental” ones, such as the frequently employed dichotomy in spatial navigation between egocentric vs. allocentric spatial information. As discussed above, however, we lack process pure measures of these codes, and must rely on tasks that are more likely to require one reference frame or the other for the optimal solution. To address this issue, we propose the RVD task that we believe utilizes more allocentric information than the commonly used JRD task. We then consider how encoding modality might influence egocentric and allocentric codes, and in particular, how we translate between them. We conclude that while studying human spatial navigation with ongoing neural recordings requires some compromise based on using fMRI/scalp EEG and desktop VR, with some expected changes in how these representations manifest, overall, these will not dramatically alter human navigation codes. Together, we hope the discussion provided here can provide useful considerations for research paradigms involving evaluating how spatial information will be acquired and deployed during encoding and retrieval from different modalities, such as desktop VR vs. real-world navigation.

## Author Contributions

MS developed these perspectives under the supervision of AE. MS drafted the manuscript and AE provided critical revisions.

## Conflict of Interest Statement

The authors declare that the research was conducted in the absence of any commercial or financial relationships that could be construed as a potential conflict of interest.
